# Wnt and Notch signaling pathway involved in wound healing by targeting *c-Myc* and *Hes1* separately

**DOI:** 10.1186/s13287-015-0103-4

**Published:** 2015-06-16

**Authors:** Yan Shi, Bin Shu, Ronghua Yang, Yingbin Xu, Bangrong Xing, Jian Liu, Lei Chen, Shaohai Qi, Xusheng Liu, Peng Wang, Jinming Tang, Julin Xie

**Affiliations:** Department of Medical Cosmetology, Jiangxi Maternal and Child Health Hospital, Eight one avenue, Nanchang, 330008 China; Department of Burns Surgery, First Affiliated Hospital of Sun Yat-Sen University, Zhongshan road, Guangzhou, 510080 China; Department of Burns, Fo Shan Hospital of Sun Yat-Sen University, Lingnan avenue, Foshan, 528000 China; Department of Burns, Third Affiliated Hospital of Sun Yat-sen University, Tianhe road, Guangzhou, 510620 China

## Abstract

**Introduction:**

Wnt and Notch signaling pathways are critically involved in relative cell fate decisions within the development of cutaneous tissues. Moreover, several studies identified the above two pathways as having a significant role during wound healing. However, their biological effects during cutaneous tissues repair are unclear.

**Methods:**

We employed a self-controlled model (Sprague–Dawley rats with full-thickness skin wounds) to observe the action and effect of Wnt/β-catenin and Notch signalings in vivo. The quality of wound repair relevant to the gain/loss-of-function Wnt/β-catenin and Notch activation was estimated by hematoxylin-and-eosin and Masson staining. Immunofluorescence analysis and Western blot analysis were used to elucidate the underlying mechanism of the regulation of Wnt and Notch signaling pathways in wound healing. Meanwhile, epidermal stem cells (ESCs) were cultured in keratinocyte serum-free medium with Jaggedl or in DAPT (N-[(3,5-difluorophenyl)acetyl]-L-alanyl-2-phenyl]glycine-1,1-dimethylethyl) to investigate whether the interruption of Notch signaling contributes to the expression of Wnt/β-catenin signaling.

**Results:**

The results showed that in vivo the gain-of-function Wnt/β-catenin and Notch activation extended the ability to promote wound closure. We further determined that activation or inhibition of Wnt signaling and Notch signaling can affect the proliferation of ESCs, the differentiation and migration of keratinocytes, and follicle regeneration by targeting *c-Myc* and *Hes1*, which ultimately lead to enhanced or delayed wound healing. Furthermore, Western blot analysis suggested that the two pathways might interact in vivo and in vitro.

**Conclusion:**

These results suggest that Wnt and Notch signalings play important roles in cutaneous repair by targeting *c-Myc* and *Hes1* separately. What’s more, interaction between the above two pathways might act as a vital role in regulation of wound healing.

## Introduction

The skin is the largest organ in the human body and stems aggression of external microorganisms and dehydration. As a response to and result of injury, several dynamic and interactive processes occur and eventually lead to wound healing, which involves regeneration of the normal structure and function of the organ. The success of the wound repair depends on the differentiation and proliferation of involved cells, including epidermal stem cells (ESCs), keratinocytes, and fibroblasts, together with the assistance of various biological signals. Moreover, these signals contribute significantly to regulate biological actions of cells within epithelial tissue. Therefore, under the incorrect guidance of signals, activities of the above cells change and the resulting wound healing is abnormal (that is, either lingering or excessive). According to increasing advances in wound-healing research, Wnt and Notch signaling pathways play a key role in the regulation of migration, proliferation, and differentiation of cells functionally relevant to skin tissue repair.

Depending on different contents, Wnt ligands (like Wnt1) signal by the canonical or non-canonical Wnt signaling pathways. For the canonical Wnt signaling pathway, β-catenin is the key mediator. When the canonical Wnt signaling is initiated, cytoplasmic and nuclear levels of β-catenin can increase and ultimately activate target genes (like *c-Myc*). Wnts are important for many fundamental cellular processes in the regulation of development and homeostasis [1]. Wnt action determines ESCs in mice to maintain the stem cell state or their entry into the differentiation stage [2], and the loss of Wnt causes ECSs to differentiate into keratinocytes and sebocytes. *c-Myc*, a downstream target of canonical Wnt/β-catenin signaling, functions as a regulator of stem cell maintenance. The downregulation of *c-Myc* can induce the depletion of ESCs in vivo [3] but can cause differentiation of ESCs in vitro [4]. On the other hand, Notch signaling is also involved in regulating cell fate; in light of different cell types and contexts, Notch signaling induces cell differentiation or maintains cells in an undifferentiated proliferation state [5]. Accompanied by Notch ligands (like jagged1) binding to Notch receptors (like Notch1), a Notch intracellular domain (NICD) can be released and translocated to the nucleus, where it modulates target genes such as Hairy and enhancer of split 1 (*Hes1*). Notch signaling has been reported to be observed in the cells of the epidermis, and blocking Notch signaling leads to the downregulation of expression of differentiation markers [6]. *Hes1* is a known target of Notch signaling and plays an important role in the maintenance of proliferating cells. When intestinal adenomas expressed *Hes1* at a low level, many tumor cells exited the cell cycle and did not continue to proliferate [7] in vivo. However, it was unclear whether *Hes1* is similarly important for regulating epidermal cells within wound healing.

Given identifications of Wnt/β-catenin and Notch signalings in skin, the application of the two pathways may be a potential avenue to improve wound healing and inhibit scar formation. However, the exact roles and underlying molecular mechanisms for the above two pathways related to wound repair are not completely clear, which undoubtedly block the exploration of the ultimate solution to both underhealing and overhealing. Therefore, the aim of this study is to observe the actions of Wnt/β-catenin and Notch signalings and to investigate effect of the two signalings for wound healing. The results of this study can offer a theoretical foundation for the treatment of lingering wound healing and excessive wound healing.

## Methods

### Ethics statement

All animal experiments were approved by the Institutional Animal Care and Use Committee at Sun Yat-Sen University and performed according to National Institutes of Health guidelines. Sprague–Dawley (SD) pregnant rats were obtained from the Experimental Animal Center of Sun Yat-Sen University and kept under standard conditions according to the regulation of ethics committee of the Medical Sciences Department.

### Wound model and wound analysis

We used a label-retaining technique to assay the number of stem cells. By definition, ESCs rarely divide and have unlimited capacity for self-renewal. Thus, 75 newborn SD rats (10–11 days old) were injected with 50 μg/g 5-bromodeoxyuridine (BrdU) (Sigma-Aldrich, St. Louis, MO, USA) four times every 12 hours to identify ESCs [8]. As ESCs possess a longer period between divisions, they should be the only cells to retain the BrdU label after the 60-day chase period. Therefore, two full-thickness skin wounds that were 2 cm in diameter and 1–1.5 cm off the midspinal line were established on the each side of the dorsum of rats (60 days old). The wound was covered with a transparent semi-occlusive dressing (Tegaderm, 3M, Saint Paul, MN, USA) to prevent desiccation. The above wounds were randomly divided into six groups. Lithium chloride (LiCl), Dickkopf-1 (DKK1), recombinant human nuclear factor-kappa-B (rhNF-κB), and DAPT (N-[(3,5-difluorophenyl)acetyl]-L-alanyl-2-phenyl]glycine-1,1-dimethylethyl) (all from Sigma-Aldrich) were topically and respectively applied to the wound under the occlusive dressing once daily until wound closure according to the requirement, allowing the solution to bathe the wound [9–11], but there was no treatment in the control group. LiCl and DKK1 are the Wnt agonist and antagonist, whereas rhNF-κB and DAPT are the Notch agonist and antagonist. LiCl is a glycogen synthase kinase 3 beta (GSK3β) inhibitor that weakens the GSK3β-mediated degradation of β-catenin through phosphorylation of GSK3β [12]. It was used in some studies to activate the canonical Wnt signaling pathway [13]. Alternately, DKK1 can bind to Wnt co-receptors LRP 5/6 and prevent the interaction of Wnt proteins and their receptors. Therefore, it was used to negatively regulate the Wnt/β-catenin signaling pathway [14]. Although rhNF-κB is not a Notch agonist, it can trigger the Notch signaling pathway by directly inducing the expression of Jagged1. DAPT, a γ-secretase inhibitor, inhibits Notch signaling by blocking the cleavage of NICD necessary for activation of transcription of downstream target genes. DAPT has also been shown in a previous study to inhibit Notch signaling [15].

Wounds were observed until closure or animals were killed. Fifteen rats were euthanized and their wounds were photographed on days 0, 7, 14, 21, and 30. The excisional wounds, together with a peripheral rim of normal skin, were excised for further study. Wounds were digitally photographed at the time of generation (day 0) and again on days 7, 14, 21, and 30 or until wound closure. The wound areas were measured by using National Institutes of Health ImageJ software and standardized by comparison with the original wound size. The residual wound area rate was calculated as [(day n area) / (day 0 area)] × 100 % (*n* = 0, 7, 14, 21, or 30).

### Histological analysis and immunofluorescence analysis

For further histological study, tissues were fixed on formaldehyde and dehydrated and embedded in paraffin and sectioned at 4 μm. The sectioned tissues were deparaffinized and stained with hematoxylin and eosin (H&E) and Masson. The skin tissue was examined in random order under blindfold conditions with standard light microscopy.

Sections were blocked in 10 % goat serum for 30 min at 37 °C. For double labeling, two compatible primary antibodies were simultaneously applied overnight at 4 °C. The following primary antibodies were applied: mouse monoclonal anti-BrdU antibody (1:50, Sigma-Aldrich) and rabbit anti-*Hes1* antibody and rabbit anti-*c-Myc* antibody (both 1:50, Bioss, Woburn, MA, USA). The samples were then incubated for 1 h in 0.01 M phosphate-buffered saline (PBS), pH 7.4, containing the following secondary antibodies: goat anti-mouse IgG labeled with Alex Fluor 488 or goat anti-mouse IgG labeled with Alexa Fluor 594 (all 1:100, Maibio, Shanghai, China). Finally, the nuclei were stained with 4’,6-diamidino-2-phenylindole (DAPI). To provide negative controls, we performed staining in the absence of the primary antibodies by adding only PBS to the sections. Sections were documented with a fluorescence microscope (Leica, Wetzlar, Germany).

### Epidermal stem cell isolation and culture

After the rats were sacrificed (fetal, 19- to 21-day gestational age), the skin from the dorsum of each rat was taken. After that, the skin tissue was successively rinsed, immersed, digested, and incubated. After peeling off the epidermis and cutting into the microskin, the skin sample was digested with 0.25 % trypsin (cat. no. SH3008742.01, HyClone, Logan, USA) to prepare a single cell suspension, which was stopped with the addition of an equal volume of high-glucose Dulbecco’s modified Eagle’s medium (DMEM). After filtering and removal of the supernatant of the cells, the cells were resuspended in high-glucose DMEM. After 15 min at 37 °C, the above ones were detected under an inverted phase-contrast microscope. The suspended cells were collected besides the adherent cells. After 24 h, the culture medium of the remaining cells was changed to keratinocyte serum-free medium (K-SFM) (cat. no. 17005042, Gibco, part of Thermo Fisher Scientific Inc., Waltham, MA, USA). When the culture reached 70 % to 80 % confluence, the cells were digested in 0.25 % trypsin at 37 °C with 5 to 10 min of oscillation and passaged at a ratio of 1:2.

### Signaling pathway analysis in epidermal stem cells

ESCs were plated in six-well plates at a density of 10,000 cells/cm^2^ in K-SFM. The medium of the cells was supplemented with either Jagged1/FC (1000 ng/mL; R&D Systems, Minneapolis, MN, USA) or DAPT for interfering with the action of the Wnt/β-catenin and Notch signaling pathway. Cells in the medium with PBS were used as the control group.

### Western blot analysis

Proteins of cell lysates or the skin homogenates were separated by 5 % SDS-PAGE electrophoresis and transferred into nitrocellulose membranes. After blocking with TBS/5 % nonfat dry milk, the membrane was incubated with the following antigens: rabbit anti-Wnt-1 antibody (Bioworld, Dublin, OH, USA), rabbit anti-β-catenin antibody (Cell Signaling Technology, Danvers, MA, USA), rabbit anti-*c-Myc* antibody (Bioss), rabbit anti-Jagged1 antibody (Bioss), rabbit anti-Notch1 antibody (Cell Signaling Technology), and rabbit anti-*Hes1* antibody (Bioss). Immunoreactive bands were visualized by peroxidase-conjugated secondary antibodies. The signal from immunoblotting bands was captured (G:BoxiChemi camera; Mshot, Guangzhou, China) and quantified by using GIS1000 software. Quantitative Western blot measurements of target protein were normalized by corresponding measures of GAPDH derived from the same samples in each blot.

### Statistical analysis

Data are expressed as the mean ± standard error of the mean. Comparisons of changes in levels of signaling component expression between control and experimental groups at the same time point were conducted by using Student’s *t* test. The differences between the groups at different time points were compared by one-way analysis of variance followed by the Bonferroni test. All statistical analyses were performed by using SPSS 20.0 software (SPSS, Chicago, IL, USA), and a *P* value of less than 0.05 was considered statistically significant.

## Results

### Effects of several disturbing reagents on Wnt/β-catenin signaling pathway and Notch signaling pathway during wound healing

**Fig. 1:**
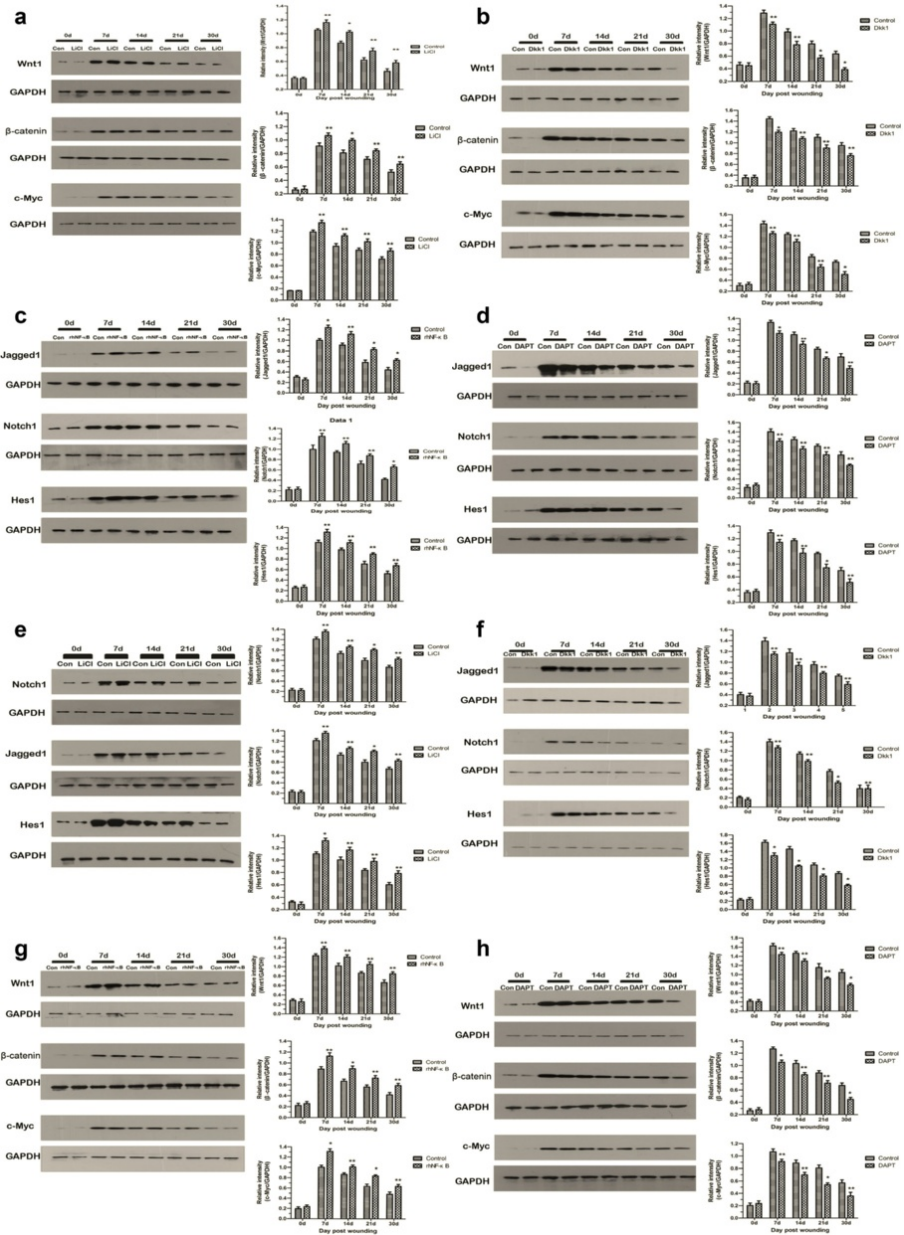
The activities of Wnt and Notch pathways and interaction were analyzed by immunoblot. Wnt activity in wound tissue is increased in response to lithium chloride (*LiCl*) treatment and is decreased in response to Dickkopf-1 (*DKK1*) (**a**, **b**). Representative immunoblot and results of densitometric analysis of blots (**a**, **b**) showed relative levels of Wnt signaling components at the indicated post-wounding time points. Notch activity in wound tissue is increased in response to recombinant human nuclear factor-kappa-B (*rhNF-κB*) treatment and is decreased in response to N-[N-(3,5-difluorophenacetyl)-L-alanyl]-S-phenylglycine t-butyl ester (*DAPT*) (**c**, **d**). Representative immunoblot and results of densitometric analysis of blots (c, d) showed relative levels of Notch signaling components at the indicated post-wounding time points. Wnt and Notch pathways interact during wound healing in wound tissue samples (**e**-**h**). Representative immunoblot and results of densitometric analysis of blots (**e**, **f**) showed relative levels of Notch signaling components from LiCl-/DKK1-treated and control rats at the indicated post-wounding time points. Representative immunoblot and results of densitometric analysis of blots (**g**, **h**) showed relative levels of Wnt signaling components from rhNF-κB-/DAPT-treated and control rats at the indicated post-wounding time points. *P < 0.01, **P < 0.05 compared with the control value (*n* = 5)

To achieve the gain- and loss-of-function studies of Wnt/β-catenin and Notch signalings during wound healing, LiCl, DKK1, rhNF-κB, and DAPT were respectively applied to the wounded skin. The disturbing reagents above were topically and respectively applied to the wound under the transparent semi-occlusive dressing (3M) once daily until wound closure in experimental groups, allowing the solution to bathe the wound. In the control group, there was no treatment. Wnt1, β-catenin, *c-Myc*, Notch1, Jagged1, and *Hes1*, as Wnt/β-catenin and Notch signaling components, were examined by Western blot to evaluate the action of two signaling pathways during the wound repair process. As shown in Fig. 1, the expression levels of two signaling components increased sharply from the start to the 7th day and then decreased from the 14th to the 30th day. Compared with the results of the control group, the results showed that the expression levels of Wnt/β-catenin signaling components were higher after in vivo LiCl treatment from the 7th to the 30th day post-burn, whereas the addition of recombinant Dkk1 resulted in lower levels (at least *P* < 0.05, Fig. 1a, b).

Similarly, when comparing relative protein values of Notch signaling components between the rhNF-κB/DAPT group and the control group, the values in the rhNF-κB group were much higher than that in the control group, and that in the DAPT group was significantly lower at the above time points (at least *P* <0.05, Fig. 1c, d). Because the high levels of Wnt1 expression and nuclear accumulation of β-catenin are defined as features of an activated Wnt/β-catenin signaling [16]. Furthermore, interaction between Notch 1 and Jagged1 can promote migration of the Notch intracellular domain (NICD) into the nucleus. NICD is the indicator of Notch pathway activation. Accordingly, the above results document the mentioned agents (LiCl, DKK1, rhNF-κB and DAPT) do affect the activity of these pathways during wound repair.

In addition, we noticed that the activation of the Wnt/β-catenin pathway promoted an upregulated protein expression of Notch components from the 7th to the 30th day but that its inhibition resulted in downregulated levels of Notch components. A similar situation appeared in the rhNF-κB group and the DAPT group at the above time points. Furthermore, the above differences were considered significant (at least *P* <0.05, Fig. 1e-h). That means a cross-talk among the above pathways may exist.

### The observation of wound healing among the groups

The roles of Wnt/β-catenin and Notch signalings in wound healing were first investigated by comparing the rate of dermal wound repair in all groups. Full-thickness skin wounds were made on each side of the dorsum of the rats and were randomly divided into the experimental groups (the LiCl group, the DKK1 group, the rhNF-κB group, and the DAPT group) and the control group. Wounds were observed until closure or animals were killed and were digitally photographed at the time of generation (day 0) and again on days 7, 14, 21, and 30 or until wound closure. The residual wound area rate was calculated as [(day n area) / (day 0 area)] × 100 % (*n* = 0, 7, 14, 21, or 30).

**Fig. 2:**
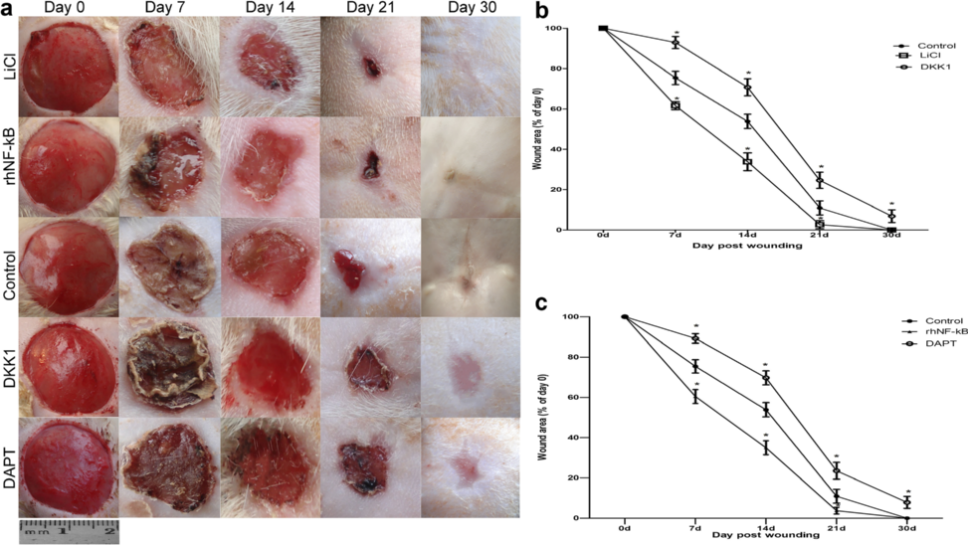
Pharmacological effect of Wnt and Notch impairs wound healing. Full-thickness dermal wounds were induced in control rats, lithium chloride (*LiCl*)-treated rats, Dickkopf-1 (*DKK1*)-treated rats, recombinant human nuclear factor-kappa-B (*rhNF-κB*)-treated rats, and N-[N-(3,5-difluorophenacetyl)-L-alanyl]-S-phenylglycine t-butyl ester (*DAPT*)-treated rats. **a** Images of a representative mouse from each group taken on post-injury days 0, 7, 14, 21, and 30 are shown. **b** Wound areas are shown for control wounds, LiCl-treated wounds, and DKK1-treated wounds. The computation was that the indicated area was divided by the initial area. Results represent mean ± standard error of the mean (*SEM*) (*n* = 10 for each group). **c** Wound areas are shown for control wounds, rhNF-κB-treated wounds, and DAPT-treated wounds. The computation was that the indicated area was divided by the initial area. Results represent mean ± SEM (*n* = 10 for each group). *P < 0.01 compared with the control value

In LiCl-/rhNF-κB-treated rats, 2-cm full-thickness dermal wounds were healed significantly, with the lesions being reduced by over 60 % within 14 days compared with control rats, and were completely healed within 30 days (Fig. 2b, c). Furthermore, gross observation of wounds indicated that rats treated with LiCl or rhNF-κB had better wound-healing quality, such as scarless healing, which is barely detected in control rats (Fig. 2a). However, healing exhibited a significant delay in DKK1-/DAPT-treated rats, compared with the control rats, with the lesion size being decreased by less than 30 % at 14 days. On day 30 after wounding, the wounds in DKK1-/DAPT-treated rats were incompletely closed (Fig. 2a).

### Wnt/β-catenin signaling pathway and Notch signaling pathway mediate hair follicle formation, re-epithelialization, and the generation and deposition of collagen during wound healing

**Fig. 3:**
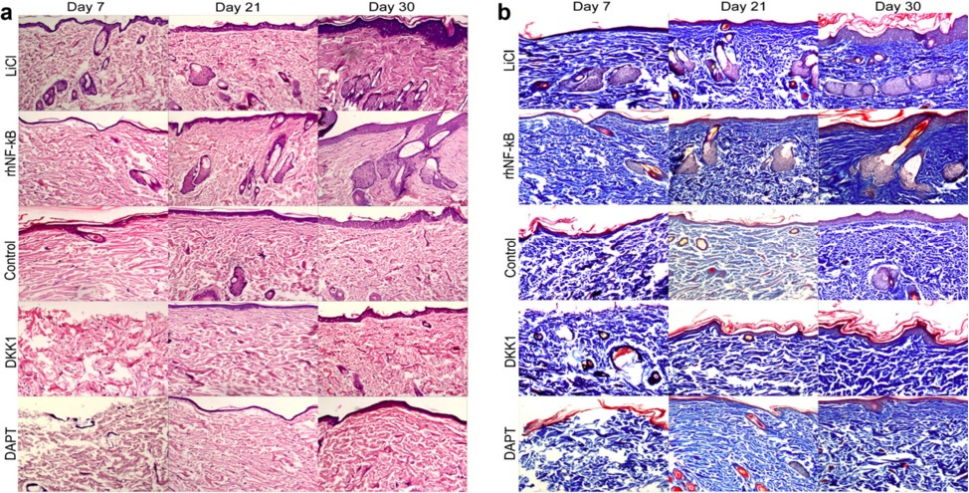
Histological features of wound healing in rats with decreased or increased Wnt and Notch activity. Images of skin tissue sections stained with hematoxylin and eosin (**a**) and Masson (**b**) showing histological changes during the wound-healing process in control, lithium chloride (*LiCl*)-treated, Dickkopf-1 (*DKK1*)-treated, recombinant human nuclear factor-kappa-B (*rhNF-κB*)-treated, and N-[N-(3,5-difluorophenacetyl)-L-alanyl]-S-phenylglycine t-butyl ester (*DAPT*)-treated rats at post-injury days 7, 14, and 30. Compared with control rats, LiCl-treated and rhNF-κB-treated rats exhibited a significant epidermal thickening, an orderly collagen, and regeneration of the skin appendages with enhanced wound healing. DKK1-treated and DAPT-treated rats exhibited delayed and poor-quality wound healing in comparison with control rats. Original magnification, 100×. Arrow pointed to neoformative epidermis layer

To further evaluate the quality of wound healing, we observed hair follicle regeneration, re-epithelialization, and the generation and deposition of collagen during cutaneous wound closure by H&E and Masson staining. Detailed histopathological analysis of relative sections showed that the neoformative epidermis layer was significantly thicker in the LiCl/rhNF-κB group than in the control group, resulting in more cell layers, more epidermal ridges, more formation of primitive hair follicle structures and sebaceous glands, and a more regular and ordered collagen arrangement (Fig. 3). Downregulation of Wnt/β-catenin and Notch signaling component expression impairs epidermis re-formation, the collagen arrangement, and skin appendage regeneration in the DKK1/DAPT group compared with the control group (Fig. 3).

Collectively, the analysis of the histopathological sections suggests that these pathways have an active help in the regulation of hair follicle re-formation, re-epithelialization, and the arrangement and deposition of collagen but that the suppression of the two pathways in vivo resulted in poor-quality wound healing.

### Wnt/β-catenin signaling pathway and Notch signaling pathway stimulate the proliferation of epidermal stem cells during wound healing

**Fig. 4:**
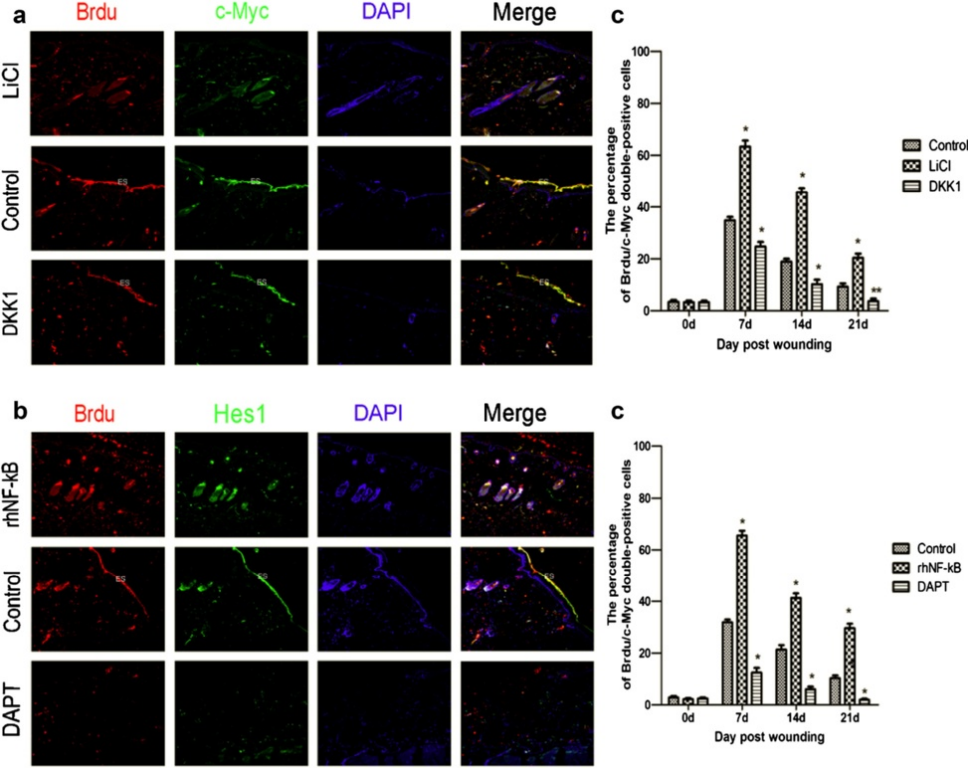
The relationships of the Wnt and Notch signaling pathway and the proliferation of epidermal stem cells was analyzed by immunofluorescence. **a** and **c**. Representative BrdU/*c-Myc* double-positive cells in wounded skin on day 7 (**a**), and the percentage of the positive cells to total cells in wound tissue at the indicated post-wounding time points (**c**). **b** and **d**. Representative BrdU/*Hes1* double-positive cells in wounded skin on day 7 (**b**), and the percentage of the positive cells to total cells in wound tissue at the indicated post-wounding time points (**d**). *P < 0.01, **P < 0.05 compared with the control value (*n* = 5). Original magnification, 100×. Scale bar = 100 μm. *BrdU* 5-bromodeoxyuridine, *DAPT* N-[N-(3,5-difluorophenacetyl)-L-alanyl]-S-phenylglycine t-butyl ester, *DKK1* Dickkopf-1, *Hes* hairy and enhancer of split, *LiCl* lithium chloride, *rhNF-κB* recombinant human nuclear factor-kappa-B

In wound repair, ESCs play a vital role by providing the source for replenishing lost cells during wound healing [17, 18]. For this, we employed BrdU, *c-Myc*, and *Hes1* as markers by double-immunofluorescence staining to detect the relationship between ESC proliferation with the two signalings. Among this, BrdU marks ESCs [3]. BrdU*/c-Myc* double-positive cells and BrdU/*Hes1* double-positive cells were bare in all groups in the normal skin tissue (Fig. 4c, d). After wounding, the number of positive cells gradually increased in response to wounding, reaching peak level on the 7th day and downregulated thereafter. The positive cells were detected in hair follicle cell nucleus and skin basal cell nucleus (Fig. 4a, b). Furthermore, the number of BrdU/*c-Myc* double-positive cells was predominantly upregulated in the LiCl group compared with that in the control culture, and that of the DKK1 group was distinctly downregulated compared with that of the control group on day 7 (Fig. 4a, b). Meanwhile, the number of BrdU/*Hes1* double-positive cells was markedly increased in the rhNF-κB group compared with the control culture, whereas that of the DAPT group was significantly decreased compared with that of the control group during wound repair (at least *P* <0.05, Fig. 4c, d). That means that Wnt/β-catenin and Notch signals could significantly stimulate the proliferation of ESCs during the wound-healing process.

### Expression changes in Wnt1, β-catenin, and *c-Myc* after Jagged1 treatment

**Fig. 5:**
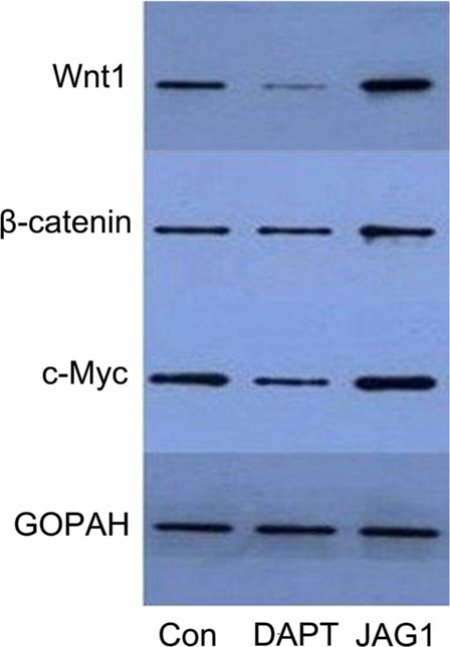
The interruption of Notch signaling contributes to the expression of Wnt/β-catenin signaling, which was detected by Western blot. When epidermal stem cells were cultured in keratinocyte serum-free medium with Jagged1 or N-[N-(3,5-difluorophenacetyl)-L-alanyl]-S-phenylglycine t-butyl ester (*DAPT*), the expression of protein in the Wnt pathway was accordingly increased or decreased. *Con* control

To investigate whether the interruption of Notch signaling contributes to the expression of Wnt/β-catenin signaling in ESCs, ESCs were cultured in K-SFM with Jaggedl or in DAPT. The changes in protein expression of Wnt1, β-catenin, and *c-Myc* after Jagged1 treatment were quantitatively analyzed by Western blot. As shown in Fig. 5, the expression levels of proteins in Wnt/β-catenin signaling were decreased after DAPT treatment and those in the Jagged1 group were significantly elevated. Because we have found that the Notch signaling pathway stimulated the proliferation of ESCs during wound healing (Fig. 4c, d), it is possible that the elevated level of Wnt activity is the accompaniment of the proliferation of ESCs. Based on these findings, it can be speculated that Wnt activity synchronizes with Notch activity in ESCs (Jag1 is also an activator for the Notch pathway). Whether Jag1 activates Wnt directly needs further study and we will try our best to study this in the future.

### Communication between the Wnt/β-catenin signaling pathway with the Notch signaling pathway during wound healing

**Fig. 6:**
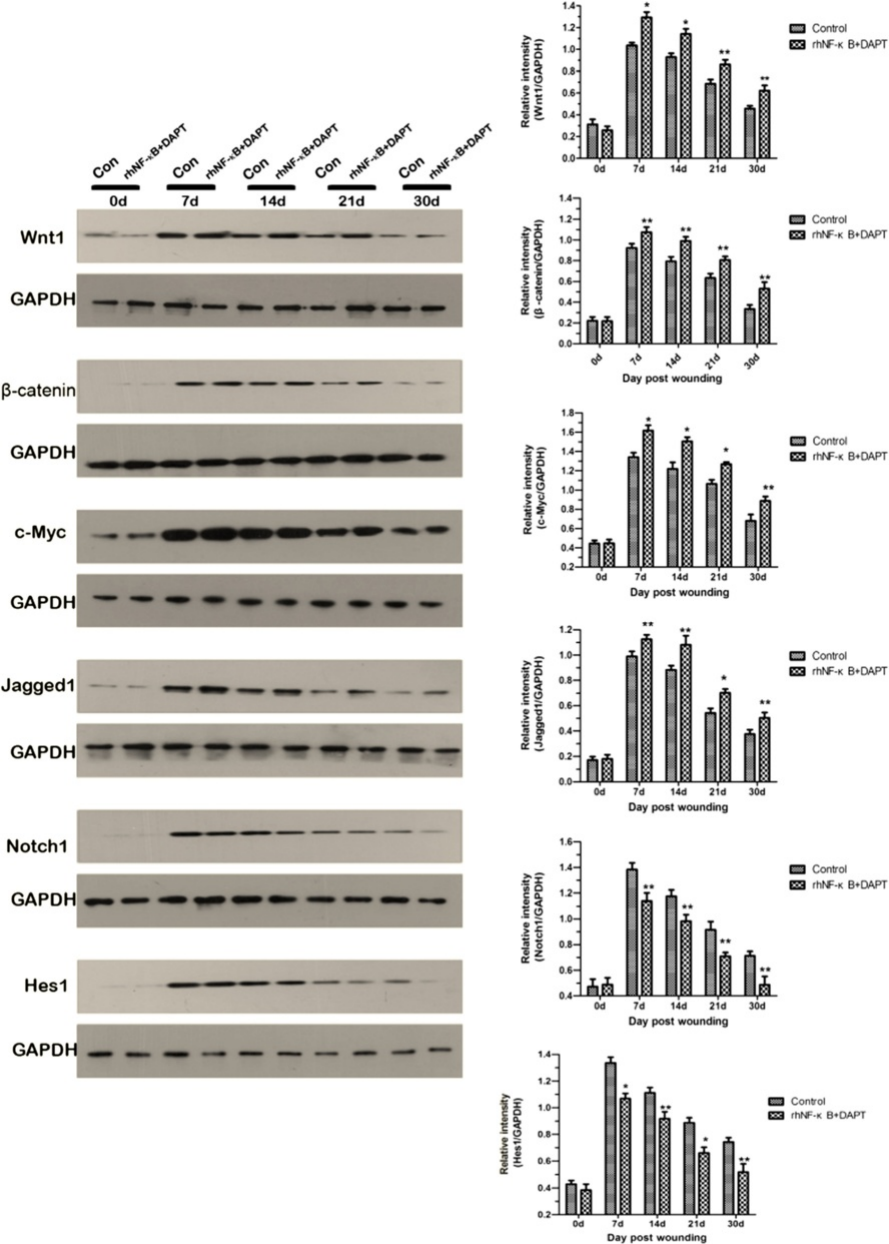
Jagged1 mediates the communication between Wnt and Notch pathways within wound repair. Representative immunoblot and results of densitometric analysis of blots showed relative levels of Wnt and Notch signaling components from recombinant human nuclear factor-kappa-B (*rhNF-κB*) and N-[N-(3,5-difluorophenacetyl)-L-alanyl]-S-phenylglycine t-butyl ester (*DAPT*)-treated and control mice at the indicated post-wounding time points. The detailed analysis indicated a significant increase in the expression of Jagged1 with a marked decrease in the expression of Notch1 and *Hes1*, whereas the components of Wnt signaling presented a pronounced increase in expression following the expression of Jagged1. *P < 0.01, **P < 0.05 compared with the control value (*n* = 5)

To further identify the communication between the Wnt signaling pathway with the Notch signaling pathway within adult mammalian wound repair, rhNF-κB and DAPT were simultaneously added into the wound tissue at the time of the experimental observations. As shown in Fig. 6, treatment with rhNF-κB and DAPT produced a significant increase in the expression of Jagged1 and a marked decrease in the expression of Notch1 and *Hes1*. Simultaneously, Wnt/β-catenin signaling components presented a pronounced increase in expression in response to the expression of Jagged1 in vivo.

## Discussion

The signaling pathways are responsible for regulating the entire wound-healing process, which includes the Wnt/β-catenin and Notch signaling pathway. Several pieces of evidence have shed light on this. The activation of the above two pathways during wound repair has been observed in our previous study [19]. In the present study, we also showed that in vivo the gain-of-function Wnt/β-catenin and Notch activation extended the ability to promote wound closure but that the inhibition of the relative pathways obviously led to delayed and poor-quality wound healing (Fig. 2). In the skin, Wnts are important mediators of wound regeneration and participate in various processes, from the development of the dermis to the formation of skin appendages [20]. Overexpression of Wnt signaling molecules promotes cellular proliferation, migration, and extracellular matrix (ECM) degradation which reflect the basic phases of wound regeneration. Similarly, the Notch signaling pathway is documented by Bielefeld et al. to be a regulator of epidermal differentiation [21]. A high level of Notch signaling activity promotes the differentiation of ESCs into keratinocyte and interfollicular lineages [22]. In view of the above findings, it is suggested that expression and activation of the above two signalings do have an active role in facilitating wound healing and reducing scarring. However, the mechanism by which the function of Wnt/β-catenin and Notch signaling pathway relevant to cutaneous wound is realized is unknown. To identify the biological functions and mechanisms of the two pathways, we detected the biological action of the cells of various types based on the gain- and loss-of-function studies of Wnt/β-catenin and Notch signaling during the entire wound-healing process.

As we all know, ESCs have an important role in wound repair [23, 24] and are considered to be the source for replenishing lost cells during wound healing. ESCs can switch rapidly and reversibly between quiescence and activity following injury or drug treatment or both [25]. In response to injury, activated ESCs can migrate to the wound site, proliferate, and differentiate into keratinocytes that reconstruct the epidermal barrier [23]. Thus, we carried out BrdU/*c-Myc* labeling and BrdU/*Hes1* labeling to determine the proliferation rate of ESCs and the interactions between ESCs and Wnt/β-catenin or Notch signaling in the cutaneous lesions. *c-Myc* and *Hes1* are respective downstream targets of Wnt/β-catenin and Notch signaling. Our results suggest (Fig. 4) that the two signalings via *c-Myc* and *Hes1* have advantages over stimulating the proliferation of ESCs during wound healing. This finding is consistent with previous work demonstrating that *c-Myc* increases proliferation [3]. The putative mechanisms are that Wnt signaling targets *c-Myc* gene, which regulates transition from G_1_ to S phases of the cell cycle [26] and shortens the epidermal stem cell cycle duration. In addition, activation of Wnt/β-catenin pathway induces the dedifferentiation of aged epidermal cells into stem cell-like cells and is accompanied by the increased expression of *c-Myc* [27]. It is worthy of note that we address Notch signaling enhance ESCs proliferative action, which is different from prior studies [28]. A possible explanation may be that Notch signaling promotes ESCs proliferative action by targeting *Hes1*, given that Notch signaling has been shown to promote proliferation and maintain the undifferentiated state in some stem cells (like melanocyte stem cells) by targeting *Hes1* [29].

To assess the ability of Wnt/β-catenin and Notch signaling in the regulation of keratinocytes, we carried out H&E staining of the lesions at several specific times after wounding. Histological analysis of the lesions reveals (Fig. 3) that augmenting the two signals appears to enhance re-epithelialization within the damaged tissue. As it is thought that *c-Myc* overexpression could promote oriented differentiation of ESCs [30]. Several scholars have demonstrated that *c-Myc* knock-out mice display hindered re-epithelialization, because of the inability of ESCs to produce daughter keratinocytes [31]. Hence, we speculate that the epithelialization-stimulating effect of Wnt/β-catenin signaling is mediated by *c-Myc*, promoting ESC differentiation into keratinocytes [4]. However, there appears to be a contradiction in the regulation of Notch signaling to re-epithelialization. Although it has been reported that activation of Notch signaling inhibited the migration of keratinocytes, many scholars also provided emerging evidence that levels of Jagged-1 and Notch1 increased during the differentiation process and might trigger keratinocyte terminal differentiation and cornification in vitro [32, 33]. Furthermore, the mentioned studies are in agreement with our results that Notch signaling accelerated re-epithelialization in skin wounds (Fig. 3). Indeed, epithelialization is the result of three overlapping keratinocyte functions: proliferation, migration, and differentiation [34]. Accordingly, considering our observation, we speculate that the enhancement of differentiation and cornification has a significant advantage over the inhibition of migration in the regulation of keratinocyte action by Notch signaling. Moreover, the canonical function of *Hes1* can promote senescence and differentiation of NHOKs (normal human oral keratinocytes) [35].

Although the loss of an adult follicle is considered permanent, full-thickness excisional wounds in rats were found to heal with de novo hair follicle regeneration and this process requires the involvement of Wnt/β-catenin and Notch signalings in our results (Fig. 3). Indeed, recent in vivo studies have demonstrated that overexpression of Wnt ligands in the epidermis increases the number of regenerated hair follicles but that inhibition of Wnt signaling after re-epithelialization completely abrogates this wounding-induced folliculogenesis [36]. Coincidentally, a high level of activated β-catenin markedly promotes ESC differentiation into hair follicle morphogenesis [37]. Besides, the inhibition of downstream target *c-Myc* significantly restrains the development of anagen hair follicles, whereas *c-Myc* activation promotes hair growth [38]. In this study, *c-Myc* expression was also observed in neoformative hair follicle cell nucleus of wounded skin (Fig. 4). Thus, we theorize that the activation of Wnt signaling after injury could achieve the promotion of follicle regeneration by inducing β-catenin to target *c-Myc*. Additionally, Notch1 is involved in regulating invagination of hair follicles into the dermis and maintaining postnatal hair homeostasis, which indicate that Notch signaling in the wound-healing process is required for hair follicle regeneration [39]. Hair follicles express low levels of Notch signaling target gene *Hes1* in the resting phase but express high levels in the anagen phase follicles [40]. Considering *Hes1* expression in neoformative hair follicles and its upregulation tightly following Notch activation (Fig. 4), we hypothesize that Notch signaling is responsible for determining hair follicle formation by promoting the transcriptional activation of *Hes1*.

In the proliferative phase of wound healing, fibroblasts produce ECM required by closing the lesion. Among this, collagen is a key component of ECM [21]. ECM acts as not only the support platform for the migration of keratinocyte in the proliferative phase but also a major determinant for the quality of wound closure and scarring in the remodeling phase marked by collagen deposits [21]. In the present study, we observed that Wnt/β-catenin signalings promoted the generation and more orderly and regular arrangement of collagen during wound healing in parallel with Notch signaling (Fig. 3). In the case of Wnt activity, relative function for the regulation of ECM formation and deposition in skin repair has already been identified. A similar study has indicated that β-catenin protein levels and transcriptional activity are elevated in dermal fibroblasts in the proliferative phase and gradually return to baseline in the remodeling phase [41]. Elevated β-catenin levels induce increased dermal collagen deposition and myofibroblast formation [42], whereas knockdown of β-catenin in Pax7-expressing cells in murine wounds leads to fewer dermal fibroblast-like cells [43]. The above mechanism was found to involve the β-catenin-dependent Wnt pathway signals [42, 44]. In turn, Outtz et al. [45] found that Notch1 hemizygous mice exhibited increased collagen deposition in healing wounds. In systemic sclerosis (SSc), activation of Notch signaling resulted in an SSc-like phenotype with increased release of collagen and differentiation of resting fibroblasts into myofibroblasts, which required the presence of Jagged1 and *Hes1* [46]. Taking our results into account (Fig. 3), we suspect that Notch is involved in collagen deposition and scar formation by regulating the transcriptional activation of *Hes1*.

Based on the gain- and loss-of-function studies of Wnt/β-catenin and Notch in the mouse models (Fig. 1), it is likely that the pathways exist to interact in regulating wound healing. In ESCs cultured in K-SFM with Jaggedl, we observed significantly elevated expression levels of Wnt/β-catenin signaling components (Fig. 5). It can be speculated that Wnt activity also synchronizes with Notch activity in ESCs. Actually, β-catenin, as an essential component of intercellular junctions, can positively regulate Jagged1 transcriptional activity, which is required by ectopic hair follicle formation in adult epidermis [39]. Besides, the cross-talk does occur between the Wnt and Notch signaling pathways in colorectal cancer, and Jagged1 is presumed to be the pathological link between these pathways in colorectal cancer [26, 47, 48]. To further identify the communication between the Wnt signaling pathway and the Notch signaling pathway within adult mammalian wound repair, rhNF-κB and DAPT were simultaneously added into the wound tissue at the time of the experimental observations. Those who need a specification is that although the direct agonist of Notch action is Jagged1 rather than NF-κB, Jagged1 expression can be upregulated specifically by transcriptional NF-κB [49]. Therefore, NF-κB can indirectly regulate the Notch pathway. Furthermore, we want to observe the relationship between individualized expression change in Jagged1 with Wnt activity. In addition, it is already reported that applying Jag1 to wound induces a similar response [32] as described in the present article. Therefore, we choose NF-κB instead of Jagged1 to activate the Notch pathway. (DAPT inhibits the promotion of the γ-secretase enzyme [50] for proteolytic cleavages of Notch1 which finally restrain transcription.) Western blot analysis did indicate that expression of Wnt/β-catenin signaling components was upregulated, coupled with the elevated levels of Jagged1 and downregulation in the expression of Notch1 and *Hes1* (Fig. 6). It has been suggested that jagged1 may play a role in the interplay between Wnt and Notch pathways in wound repair. But current evidence is not adequate to directly document jagged1 active Wnt pathway. Besides, it is still unknown that whether NF-κB also acts through other mechanisms (not Notch activation) to activate Wnt pathway. Thus, we will make more efforts to explore and document the role of Jagged1 in the regulation of Wnt/β-catenin and Notch signaling in skin in future studies.

## Conclusions

This report provides new evidence for the potential function of Wnt and Notch signalings in regulating wound repair and their interaction. But our work has just begun. The communication among them is complex, and the mechanism that mediates Wnt and Notch pathways remains unclear. Further research is needed to elucidate the mechanism of Notch/Wnt interaction in cutaneous repair, which allows the development of new therapeutic strategies for delayed healing and pathological scarring.

## Abbreviations

BrdU: 5-bromodeoxyuridine
DAPT: N-[N-(3,5-difluorophenacetyl)-L-alanyl]-S-phenylglycine t-butyl ester
DKK1: Dickkopf-1
DMEM: Dulbecco’s modified Eagle’s medium
ECM: Extracellular matrix
ESC: Epidermal stem cell
GSK3β: Glycogen synthase kinase 3 beta
H&E: Hematoxylin and eosin
Hes: Hairy and enhancer of split
K-SFM: Keratinocyte serum-free medium
LiCl: Lithium chloride
NF-κB: Nuclear factor-kappa-B
NICD: Notch intracellular domain
PBS: Phosphate-buffered saline
rhNF-κB: Recombinant human nuclear factor-kappa-B
SD: Sprague–Dawley
SSc: Systemic sclerosis
