# Erratum to: Wnt and Notch signaling pathway involved in wound healing by targeting *c-Myc* and *Hes1* separately

**DOI:** 10.1186/s13287-015-0265-0

**Published:** 2015-12-18

**Authors:** Yan Shi, Bin Shu, Ronghua Yang, Yingbin Xu, Bangrong Xing, Jian Liu, Lei Chen, Shaohai Qi, Xusheng Liu, Peng Wang, Jinming Tang, Julin Xie

**Affiliations:** Department of Burns Surgery, First Affiliated Hospital of Sun Yat-Sen University, Zhongshan Road, Guangzhou, 510080 China; Department of Burns, Fo Shan Hospital of Sun Yat-Sen University, Lingnan Avenue, Foshan, 528000 China; Department of Burns, Third Affiliated Hospital of Sun Yat-sen University, Tianhe Road, Guangzhou, 510620 China

## Erratum

After publication of this article [1] we were informed that the affiliation for Dr Yan Shi was incorrectly listed as Department of Medical Cosmetology, Jiangxi Maternal and Child Health Hospital. Dr Shi’s correct affiliation should have been given as Department of Burns Surgery, First Affiliated Hospital of Sun Yat-Sen University. Dr Shi’s affiliation has been corrected here.

Please note that the email address for Dr Bin Shu has changed since the publication of our original article. The correct e-mail address is shubin29@sina.com.
